# Preparation of Soybean Dreg-Based Biochar@TiO_2_ Composites and the Photocatalytic Degradation of Aflatoxin B_1_ Exposed to Simulated Sunlight Irradiation

**DOI:** 10.3390/toxins16100429

**Published:** 2024-10-05

**Authors:** Jian Zhang, Zhiwei Ying, He Li, Xinqi Liu, Dongge Ma, Hailong Yu

**Affiliations:** 1 Department of Nutrition and Health, China Agricultural University, Beijing 100193, China; tsnpzhj@163.com; 2 School of Food and Health, Beijing Technology and Business University, Beijing 100048, China; yingzhiwei0906@163.com (Z.Y.); lihe@btbu.edu.cn (H.L.); 3 National Soybean Processing Industry Technology Innovation Center, Beijing Technology and Business University, Beijing 100048, China; 4 Department of Chemistry, College of Chemistry and Materials Engineering, Beijing Technology and Business University, Beijing 100048, China; 5 College of Bioengineering, Beijing Polytechnic, Beijing 100176, China

**Keywords:** photocatalyst, aflatoxin B_1_, degradation, biochar, reduction, simulated sunlight

## Abstract

Aflatoxin B_1_ (AFB_1_) is a highly toxic carcinogen severely harmful to humans and animals. This study fabricated SDB-6-K-9@TiO_2_ composites via the hydrothermal synthesis method to reduce AFB_1_. The structural characterization results of the photocatalytic composites showed that TiO_2_ was successfully loaded onto SDB-6-K-9. The different photocatalytic degradation conditions, photocatalyst kinetics, recycling performance, and photocatalytic degradation mechanism were investigated. Photocatalysis with 6 mg of 4%SDB-6-K-9@TiO_2_ in a 100 μg/mL AFB_1_ solution presented a reduction of over 95%, exhibiting excellent performance, high stability, and reusability even after five cycles of photocatalytic experiments. Active species trapping experiments confirmed that holes (h^+^) played the most critical role. After structural analysis and identification of the photocatalytic degradation products, the photodegradation path and photocatalytic oxidation mechanism of 4%SDB-6-K-9@TiO_2_ were postulated. The results show a new way to improve TiO_2_’s photocatalytic performance, providing a certain theoretical basis for the effective AFB_1_ reduction.

## 1. Introduction

Mycotoxin contamination is a significant global safety issue. Aflatoxin is a secondary metabolite produced by fungi and a harmful biological pollutant. Due to its high toxicity, teratogenicity, carcinogenicity, and mutagenicity, aflatoxin B_1_ (AFB_1_) has been listed as one of the strongest carcinogens by the International Agency for Research on Cancer (IARC) [1]. AFB_1_ poses a considerable threat to human health and the environment by spreading via the food chain. Therefore, the effective removal of aflatoxin presents a challenge for the development of the food industry. Traditional mycotoxin detoxification strategies are primarily divided into physical, chemical, and biological methods [2]. Although these techniques can remove aflatoxin to a certain extent, they remain limited. Since physical methods like adsorption and ultraviolet irradiation are simple to execute, they have been widely used in practical applications [3,4,5]. However, adsorption methods cannot decompose AFB_1_ effectively to reduce its impact on the environment and human health, while long-term ultraviolet irradiation may destroy the nutritional composition of food, resulting in safety issues [6]. Moreover, precise quantification and analysis of AFB_1_ are crucial, with the main detection methods currently including chromatographic techniques, spectrophotometry, and the combination of electrochemical immunosensing with chromatography [7], providing effective means for the detection and analysis of AFB_1_. Therefore, the increasing awareness of food safety and environmental pollution necessitates the development of efficient, feasible aflatoxin degradation technology.

Studies have demonstrated the efficacy of photocatalysis in removing organic pollutants due to its strong operational feasibility, the absence of secondary pollution, environmental friendliness, and cost efficiency. Consequently, it shows potential as an effective alternative for AFB_1_ degradation [8,9]. However, the disadvantages of low photocatalytic efficiency and an unclear mechanism significantly limit the extensive application of photocatalytic technology. As a typical semiconductor material, titanium dioxide (TiO_2_) plays an important role in the photocatalytic degradation of organic pollutants due to its strong oxidation ability, high chemical stability, and low cost [10]. However, the wide gap of TiO_2_ (rutile type = 3.0 eV, anatase type = 3.2 eV), fast electron-hole (h^+^) recombination, poor absorbability, and low visible light utilization limit its practical application [11]. Since sunlight contains only about 5% ultraviolet light, TiO_2_ is incapable of photocatalytic activity in sunlight energy exceeding 95% [12].

Extensive research has investigated new photocatalytic composites to resolve these problems, combining two or more materials to reduce the photoexcitation energy and improve photocatalytic performance. The immobilization of TiO_2_ on porous materials such as magnetic materials, zeolites, and clay can overcome some of the above shortcomings [13]. The effective loading of metal oxides on the surfaces of TiO_2_ particles can reduce the band gap [14], while non-metallic C and N element loading can change the energy level structure and the formation of new doped energy levels [15,16,17]. C loading is more successful in reducing the band gap and increasing the safety of most porous composite materials than metal oxide and N loading.

TiO_2_ composites with carbon-based materials (active carbon (AC), graphene, graphene oxide (GO), reduced graphene oxide (rGO), and multi-walled carbon nanotubes (MWCNT)) can stimulate electron transfer and exhibit better photocatalytic degradation activity [18,19,20,21]. Zhang et al. synthesized TiO_2_/pBC supported on a reed straw biochar (acid pretreated) using a sol–gel method and demonstrated, in heterogeneous photocatalysis towards sulfamethoxazole (SMX), stable photocatalytic activity [22]. Peñas-Garzón et al. investigated the degradation of three emerging pollutants (acetaminophen, ibuprofen, and antipyrine) in water using different catalysts with TiO_2_/AC heterostructures in simulated sunlight. The results showed that ibuprofen was the easiest to remove and disappeared completely within 3 h [23]. Zhu et al. prepared three different photocatalysts, indicating that Congo red displayed the best removal performance in simulated sunlight and visible light irradiation [13]. Sun et al. synthesized magnetic GO/TiO_2_ (MGO/TiO_2_) nanocomposites to reduce AFB_1_ in corn oil, which reached 96.4% after UV-vis irradiation for 120 min [21]. Compared with other carbon-based materials, the weak conductivity of AC may affect photoelectron transfer efficiency. However, its extensive specific surface area, excellent pore structure, and abundant organic functional groups on the surface effectively promote organic pollutant absorption and stimulate photocatalytic oxidation [24]. Biochar is an excellent adsorbent and catalyst carrier for removing organic pollutants because of its excellent properties similar to AC [25]. It effectively improves electron transport via dense carbon layers, oxidative and reductive groups for electron storage, charge separation, and active surface group sites, promoting the synergistic effect of adsorption and photocatalysis. The important applications of biochar as an electrochemical enhancer and anchoring system in immunosensors also suggest that its structural characteristics, conductivity, and electron transfer capabilities hold potential for environmental remediation and the removal of organic pollutants [26,27]. Biochar can be prepared from almost any biomass, including organic raw materials or waste, providing an effective method for extensive organic matter utilization.

This study used the prepared SDB-6-K-9 as a carrier to synthesize SDB-6-K-9@TiO_2_ composites via hydrothermal synthesis. The morphological, structural, and optical properties of the SDB-6-K-9@TiO_2_ composites were examined. This research also investigated the effect of different SDB-6-K-9@TiO_2_ composites, photocatalytic dosage, initial AFB_1_ concentration, and irradiation time on the photocatalytic impact of AFB_1_. The photocatalytic degradation kinetics and products were analyzed, and the photocatalytic degradation pathway and AFB_1_ mechanism were postulated.

## 2. Results and Discussion

### 2.1. The Photocatalytic Characteristics

**Figure 1:**
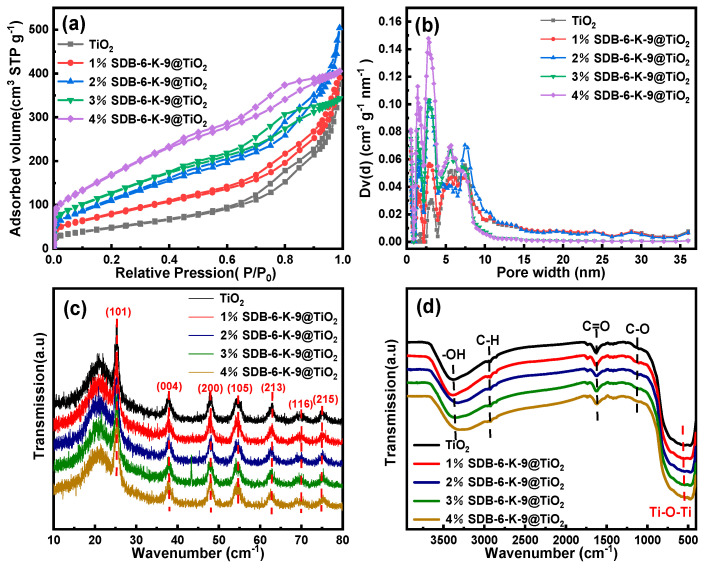
(**a**) The adsorption/desorption isotherms. (**b**) The pore size distribution maps of the TiO_2_ and SDB−6−K−9@TiO_2_ composites. (**c**) The XRD patterns. (**d**) The FTIR diagrams of the TiO_2_ and SDB−6−K−9@TiO_2_ composites.

The N_2_ adsorption–desorption isotherms of the TiO_2_ and SDB-6-K-9@TiO_2_ composites are shown in Figure 1a. From the shape, it can be concluded that the TiO_2_ and SDB-6-K-9@TiO_2_ composites present type IV isotherms and H1 hysteresis loops. A distinct hysteresis curve was evident at a relative pressure of 0.5 < P/P_0_ < 1.0, indicating the presence of mesopores. Furthermore, 4%SDB-6-K-9@TiO_2_ displayed the highest nitrogen adsorption and desorption capacity, indicating that it had the largest specific surface area. As shown in Figure 1b, the pore size distribution of the TiO_2_ and SDB-6-K-9@TiO_2_ composites were mainly concentrated in a range of 1 nm–10 nm, which was consistent with the isothermal adsorption and desorption curve analysis results, indicating the presence of microporous and mesoporous structures.

The parameter values of the specific surface area, pore volume, and average pore size are listed in Table 1. TiO_2_ had a specific surface area of 174.909 m^2^/g, a pore volume of 0.5812 cm^3^/g, and an average pore size of 1.329 nm, indicating an excellent pore structure. At a higher SDB-6-K-9 percentage, the surface area and pore volume also increased, but decreased at a higher TiO_2_ percentage. This was consistent with the decline previously observed in other biocomposites [28,29]. The specific surface area increased from 294.568 m^2^/g to 642.644 m^2^/g, while the pore volume increased from 0.5313 cm^3^/g to 0.7799 cm^3^/g. Moreover, the average pore size of the SDB-6-K-9@TiO_2_ composite was large enough to absorb AFB_1_ molecules (the molecular size of AFB_1_: length of the three sides, 1.2620 nm, 1.0968 nm, and 0.5891 nm) [30,31]. The results show that effectively combining SDB-6-K-9 and TiO_2_ promoted pore structure development without changing the original multistage pore structure.

**Figure 2:**
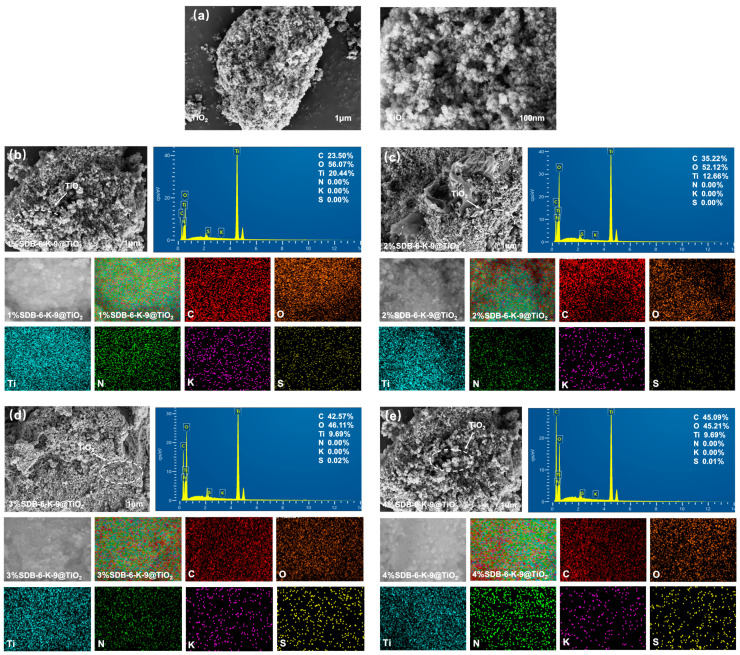
(**a**) SEM images of TiO_2_; SEM-EDS and mapping photos of (**b**) 1%SDB-6-K-9@TiO_2_; (**c**) 2%SDB-6-K-9@TiO_2_; (**d**) 3%SDB-6-K-9@TiO_2_; (**e**) 4%SDB-6-K-9@TiO_2_.

The apparent structural morphology of the TiO_2_ and SDB-6-K-9@TiO_2_ composite is shown in Figure 2. As shown in Figure 2a, the TiO_2_ prepared via hydrothermal synthesis presented an irregular spherical granular shape. Figure 2b–e indicates that TiO_2_ was uniformly supported on the surface of SDB-6-K-9. TiO_2_ particles displayed good dispersibility and particle shape retention. However, local TiO_2_ particle aggregation was also observed to some extent [32]. The elemental composition of the SDB-6-K-9@TiO_2_ composites was analyzed via EDS, and it confirmed the presence of primarily C, O, and Ti. Compared with the different addition SDB-6-K-9 amounts, the relative C content in the EDS spectra of the SDB-6-K-9@TiO_2_ composites increased. The dominant elements in TiO_2_ were Ti and O, indicating that TiO_2_ was successfully loaded onto the SDB-6-K-9 surface. The presence of C, O, and Ti during EDS mapping corresponded with the uniform distribution on the SDB-6-K-9@TiO_2_ composite surfaces.

The XRD patterns of the TiO_2_ and SDB-6-K-9@TiO_2_ composites are shown in Figure 1c. Prominent diffraction peaks were observed at 25.3°, 38.0°, 48.0°, 54.6°, 62.9°, 69.1°, and 75.0°, which could be indexed to the (101), (004), (200), (105), (213), (116), and (215) crystal planes, respectively, of the anatase-type TiO_2_ [33]. Compared with the anatase TiO_2_ standard card (JCPDS No.21-1272), the diffraction peaks were smooth and sharp with high peaks. It showed that anatase TiO_2_ prepared via hydrothermal synthesis displayed a higher purity and degree of crystallization [34]. Of the existing TiO_2_ crystalline structures (anatase, brookite, and rutile), the anatase phase exhibited the highest catalytic activity [35]. Anatase TiO_2_ diffraction peaks were evident in the SDB-6-K-9@TiO_2_ composites, indicating that the TiO_2_ was successfully loaded onto the surface of SDB-6-K-9, which was consistent with the SEM and EDS analysis results.

The FTIR spectra revealed the functional groups on the TiO_2_ and SDB-6-K-9@TiO_2_ composite surfaces. As shown in Figure 1d, compared with the FTIR spectra of SDB-6-K-9 in the previous study [36], except for the four characteristic absorption peaks of -OH, C-H, C=O, and C-O in SDB-6-K-9, a new broad absorption peak appeared at 500 cm^−1^ in the spectra of TiO_2_ and SDB-6-K-9@TiO_2_ composites had been identified, which could be attributed to the characteristic absorption peak of Ti-O-Ti tensile vibration [37]. To a certain extent, these results verified the successful loading of TiO_2_ onto the SDB-6-K-9 surface during the SDB-6-K-9@TiO_2_ composite preparation to form a new titanium-containing functional group, which was consistent with the XRD analysis.

XPS was used to characterize the surface elements and states of the SDB-6-K-9@TiO_2_ composites. The C 1s peak, Ti 2p peak, and O 1s peaks of the SDB-6-K-9@TiO_2_ composites were distinctly evident in the full survey spectra in Figure S1a,e,i,m. The primary elements, C, O, Ti, and a trace amount of the N element, were present in the samples. With the additional SDB-6-K-9 amount increasing from 1% to 4%, the C content increased from 28.13% to 56.07%, while O and Ti levels decreased from 46.82% to 29.05% and from 23.95% to 14.00%, respectively, which was consistent with the EDS elemental analysis results. The high-resolution XPS spectra of C1s in Figure S1b,f,j,n shows that the C1s curve displayed four absorption peaks around 283.8 eV, 284.9 eV, 286.3 eV, and 288.7 eV, corresponding to Csp^2^, Csp^3^, C-O, O-C=O, respectively. In Figure S1c,g,k,o, the O1s high-resolution XPS spectra exhibited two peaks at 530.0 eV and 531.6 eV, corresponding to Ti-O and -OH, respectively. The Ti2p high-resolution XPS spectra are shown in Figure S1d,h,l,p. The Ti2p curve peaks at 710.0 eV and 458.7 eV, corresponded to Ti^4+^ [38]. The existing form variation could be ascribed to specific differences in the added SDB-6-K-9 amount. However, TiO_2_ was successfully supported on the surface of SDB-6-K-9, which was consistent with the SEM-EDS, FTIR, and XRD analysis results.

**Figure 3:**
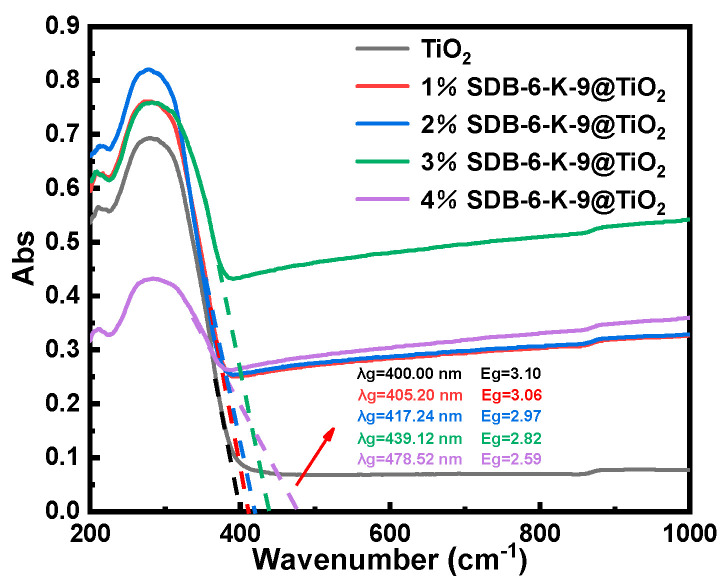
The UV-vis DRS of the TiO_2_ and SDB-6-K-9@TiO_2_ composites.

The optical properties of the TiO_2_ and SDB-6-K-9@TiO_2_ composites were examined via UV-vis spectroscopy. According to the spectral data, the absorption wavelength threshold λg was obtained using the transect method, while the excitation energy (Eg) was calculated via the formula Eg = 1240/λg (eV). As shown in Figure 3, the absorption wavelength threshold of the TiO_2_ prepared via hydrothermal synthesis was 400 nm, and the Eg value was 3.10 eV. No absorbance was evident when the wavelength exceeded 400 nm. Compared with pure TiO_2_, the absorption edge of the SDB-6-K-9@TiO_2_ composites displayed an absorbance ranging from 400 nm to 1000 nm and an obvious redshift. The Eg of the 1%SDB-6-K-9@TiO_2_, 2%SDB-6-K-9@TiO_2_, 3%SDB-6-K-9@TiO_2,_ and 4%SDB-6-K-9@TiO_2_ was 3.06 eV, 2.97 eV, 2.82 eV, and 2.59 eV, respectively. Therefore, all of the SDB-6-K-9@TiO_2_ composites displayed photoexcitation under visible light irradiation due to the presence of SDB-6-K-9, while 4%SDB-6-K-9@TiO_2_ was the most efficient. The results indicated that the excitation energy of TiO_2_ loaded with SDB-6-K-9 was effectively reduced while enhancing visible light absorption and utilization and improving photocatalytic activity [39].

### 2.2. Degradation Studies

**Figure 4:**
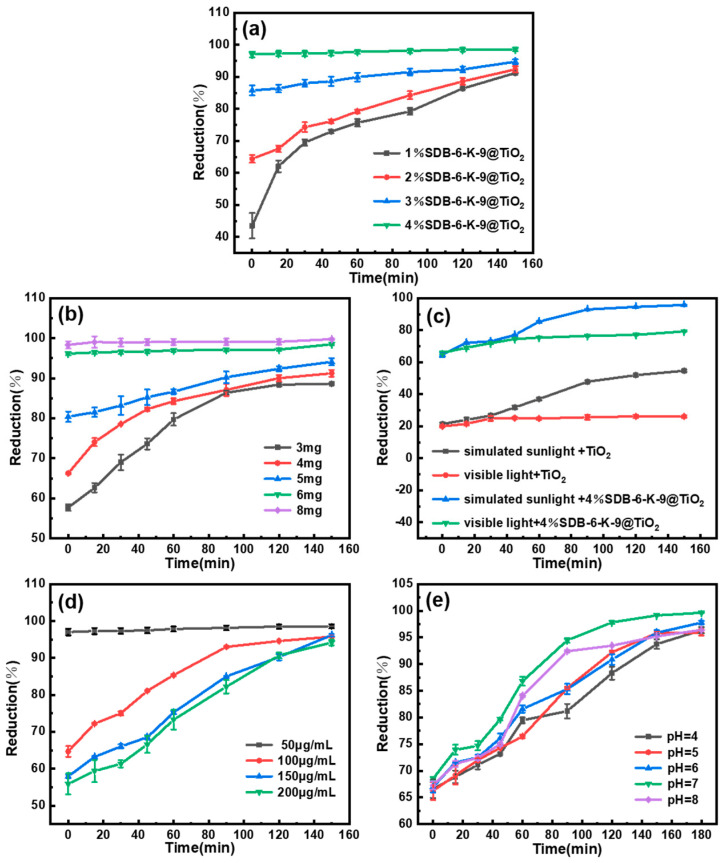
The influence of different conditions on the reduction effect. (**a**) The different photocatalysts. (**b**) The photocatalytic dosage. (**c**) The different light source. (**d**) The initial AFB_1_ concentration. (**e**) pH. The data are expressed as the mean ± standard deviation (n = 3).

The AFB_1_ degradation by the SDB-6-K-9@TiO_2_ composites in different treatment conditions is presented in Figure 4. Figure 4a shows the effect of the photocatalysts prepared by adding different amounts of SDB-6-K-9 to the SDB-6-K-9@TiO_2_ composites on the photocatalytic activity. Throughout the degradation process, all time points demonstrated that higher dosages of SDB-6-K-9 led to greater AFB_1_ reduction by the SDB-6-K-9@TiO_2_ composites. Therefore, the photocatalytic efficacy of the @TiO_2_ composite composites increased at a higher SDB-6-K-9 addition amount. The photocatalytic effect of the @TiO_2_ composites increased at a higher SDB-6-K-9 addition amount. This may be because doping SDB-6-K-9 with a high specific surface area and porosity increased the specific surface area of the SDB-6-K-9@TiO_2_ composites, providing more active sites on the surface and allowing for rapid AFB_1_ adsorption to the photocatalytic surface. The SDB-6-K-9@TiO_2_ composites, with TiO_2_ as the main active substance for photocatalysis, showed an excellent removal ability for AFB_1_ solution concentrations, with a highest removal rate of 98.57%. Therefore, 4%SDB-6-K-9@TiO_2_ was determined as the optimal photocatalyst and was selected for further photocatalytic performance tests.

The photocatalytic amount is a crucial factor affecting photocatalysis. As shown in Figure 4b, at a 4%SDB-6-K-9@TiO_2_ dose of 3 mg–8 mg, adsorption differences were evident in dark conditions. The adsorption increased at higher photocatalytic doses, probably because the abundant active sites on the surface increased at a higher 4%SDB-6-K-9@TiO_2_ dosage, promoting its adsorption effect. In the same irradiation conditions, the reduction ranged from 88.62% to 98.49% when the photocatalytic dosage increased from 3 mg to 6 mg. However, an excessive photocatalytic quantity caused TiO_2_ aggregation on the SDB-6-K-9 surface, affecting the porosity and reducing the photocatalytic activity. Therefore, 6 mg 4%SDB-6-K-9@TiO_2_ was determined as the optimal photocatalytic amount and selected for further photocatalytic performance tests.

To investigate the impact of a light source on the photocatalytic effect, TiO_2_ and 4%SDB-6-K-9@TiO_2_ were examined in simulated sunlight (300–1000 nm) and visible light (420–800 nm), respectively. The results are shown in Figure 4c. After adsorption in the dark for the first 2 h, the 4%SDB-6-K-9@TiO_2_ showed a good adsorption effect, with adsorption rates exceeding 60%, while the adsorption removal rate was only about 20% due to the pore structure of TiO_2_. With the extension of irradiation time, 4%SDB-6-K-9@TiO_2_ and TiO_2_ both exhibited good photocatalytic activity in simulated sunlight conditions with a reduction of 95.77% and 54.70%, respectively. Conversely, in visible light conditions, the reduction of 4%SDB-6-K-9@TiO_2_ and TiO_2_ only reached 79.04% and 25.57%, respectively. The results indicate that the visible light utilization and photocatalytic activity could be improved by SDB-6-K-9 loading onto the TiO_2_ surface, which was consistent with the UV-vis analysis results. Therefore, simulated daylight was determined as the optimal light source and selected for further photocatalytic performance tests.

The effect of the initial AFB_1_ concentration on the photocatalytic effect was analyzed in optimal photocatalytic conditions. As shown in Figure 4d, the reduction decreased with the initial AFB_1_ concentration from 50 μg/mL to 200 μg/mL, possibly since the increased AFB_1_ concentration occupied the effective active site on the surface. The reduction exceeded 95% with extended irradiation time. Therefore, 100 μg/mL was determined as the optimal initial AFB_1_ concentration for practical applications and selected for further photocatalytic performance tests.

The impact of pH on the photocatalytic effect was discussed according to the AFB_1_ properties, the associated charges on the surfaces of the catalyst and degraded substances, and the AFB_1_ adsorption on the photocatalytic surface. The results are shown in Figure 4e. During the degradation process, all time points indicated that the 4% SDB-6-K-9@TiO_2_ at pH = 7 achieved higher AFB_1_ reduction rates compared to acidic or alkaline conditions. Furthermore, with prolonged exposure time, the AFB_1_ reduction consistently exceeded 95%. Therefore, the 4%SDB-6-K-9@TiO_2_ photocatalyst exhibited excellent adsorption and photocatalytic degradation ability at different pH values. The absorption and degradation capacity in neutral solutions were slightly higher than in weakly acidic solutions, which was consistent with a previous report [40]. The strong AFB_1_ adsorption on the surface of the photocatalyst may be responsible for the enhanced degradation efficiency, which is probably related to the surface charge properties of the catalyst and substrate. At a pH level below 5, both the catalyst and AFB_1_ were positively charged, since the zero charge points of the TiO_2_ (pH = 6.5) and AFB_1_ (pH = 5) were higher than the pH value of the solution, leading to a certain repulsive force between the catalyst and AFB_1_ and relatively low AFB_1_ adsorption at the active catalytic site [40,41].

### 2.3. Photocatalytic Kinetics

To gain insight into the photocatalytic degradation of 100 μg/mL AFB_1_ by 4%SDB-6-K-9@TiO_2_, the Langmuir–Hinshelwood (L-H) kinetic model (ln(C_0_/C_t_) = kt, where C_0_ is the initial concentration, C_t_ is the concentration at time t, k is the reaction rate constant, respectively, was used to fit the experimental data. As shown in Figure S2, the photocatalytic AFB_1_ degradation fits the pseudo-first-order kinetic model with R^2^ values between 0.9469 and k of 0.01586.

### 2.4. Recycling Performance

Photocatalysts are difficult to separate and recycle in practical applications. To evaluate the stability and reusability of the 4%SDB-6-K-9@TiO_2_ photocatalyst, five cycles of AFB_1_ photocatalytic degradation experiments were performed in optimal conditions. Figure S3 shows the AFB_1_ degradation efficiency during various cycles. Although the photocatalytic activity of 4%SDB-6-K-9@TiO_2_ marginally decreased after multiple photocatalytic tests, the reduction remained higher than 85% after five cycles, indicating that the prepared photocatalyst displayed excellent reusability. The cause of the lower reduction rate may be related to the loss during the recovery and washing of the photocatalyst or a decline in the photocatalytic degradation rate.

### 2.5. The Photocatalytic Degradation Mechanism

The AFB_1_ photocatalytic degradation mechanism was examined via a free radical trapping experiment. In optimal photocatalytic conditions, h^+^ scavenger EDTA, hydroxyl radical (•OH) scavenger TBA, and O_2_^•−^ scavenger BQ were added to the AFB_1_ solution for the photocatalytic degradation test. As shown in Figure S4, the addition of the h^+^ scavenger EDTA significantly reduced the photocatalytic efficiency of AFB_1_ compared to the contrast group. In contrast, the inclusion of the O_2_^•−^ scavenger BQ and •OH scavenger TBA had a minimal impact on the photocatalytic efficiency of AFB_1_, indicating that they are not the primary factors influencing the degradation process. According to the findings, h^+^ played the most important role in the photocatalytic degradation process, followed by •OH, while O_2_^•−^ had a negligible impact [41].

**Figure 5:**
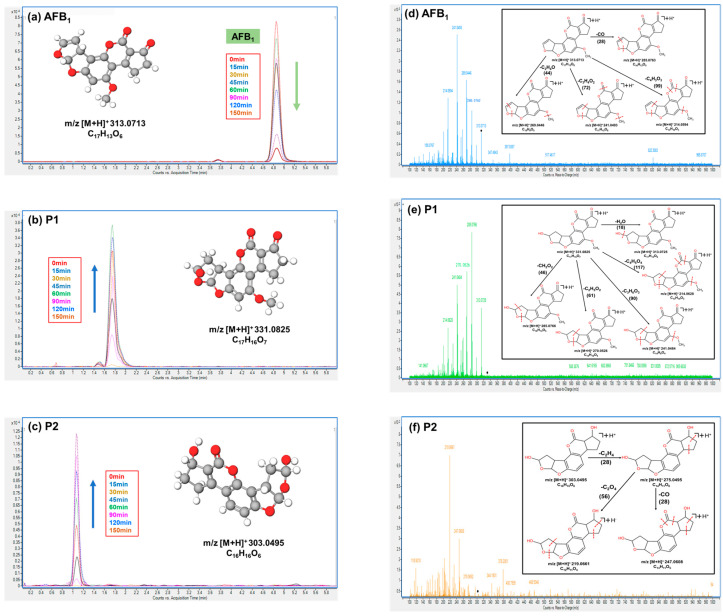
The chromatograms of the AFB_1_ and the degradation products via 4%SDB-6-K-9@TiO_2_ at different irradiation times. (**a**) AFB_1_. (**b**) The P1 photocatalytic degradation product. (**c**) The P2 photocatalytic degradation product. The TOF MS/MS spectra and possible fragmentation of the AFB_1_ and degradation products via 4%SDB-6-K-9@TiO_2_. (**d**) AFB_1_. ◆ represents the molecular weight of AFB_1_ (313.0713). (**e**) The P1 photocatalytic degradation product. ◆ represents the molecular weight of P1 (331.0825). (**f**) The P2 photocatalytic degradation product. ◆ represents the molecular weight of P2 (303.0495).

Additionally, based on the photocatalytic degradation of AFB_1_ by 4% SDB-6-K-9@TiO_2_ under pH = 7 conditions over a time course of 0 to 150 min, as shown in Figure 4e, the degradation products of AFB_1_ post-photocatalysis were further evaluated using UHPLC-Q-TOF-MS. As shown in Figure 5a–c, the AFB_1_ absorption peak (*m*/*z* 313.0713) at 4.839 min progressively diminished with the extension of light time, while new peaks developed at 1.750 min (*m*/*z* 331.0825) and 1.085 min (*m*/*z* 303.0495) and their concentrations steadily increased.

As shown in Figure 5d–f, the molecular weights of the three substances are AFB_1_ (*m*/*z* 313), P1 (*m*/*z* 331), and P2 (*m*/*z* 303). The measured molecular core of AFB_1_ is consistent with its actual molecular weight, confirming that the two newly generated degradation products are P1, with a molecular weight of 331, and P2, with a molecular weight of 303. To further identify the structures of these degradation products, AFB_1_ and the two degradation products were analyzed using UHPLC-Q-TOF-MS. The molecular nuclear ratio of the three substances were selected as the precursor ions, and secondary mass spectrometry was performed with appropriate collision energies. The MassLynx Version 4.1 software calculated the precision molecular nuclear ratio, the molecular formula with the highest match, the error, the double bond equivalents (DBE), and the i-FIT (Norm) match factor. The results show that the differences between the exact molecular masses of the three substances and their theoretical values were 0.3, 2.1, and −0.3 ppm, respectively, all within the internationally accepted standard of 5 ppm. The DBE values were 10.5, 12, and 11, with an i-FIT (Norm) value of 0 for all substances. Additionally, the molecular weight of AFB_1_ (313.0713) and its molecular formula (C_17_H_13_O_6_) as calculated by the software were consistent with its actual molecular weight (313.0712) and molecular formula (C_17_H_13_O_6_). Similarly, for P1, the molecular weight (331.0825) and molecular formula (C_17_H_15_O_7_) calculated by the software closely matched the actual values (331.0818 and C_17_H_15_O_7_). For P2, the molecular weight (303.0495) and molecular formula (C_16_H_15_O_6_) were also consistent with the actual values (303.0496 and C_16_H_15_O_6_). These results provide strong evidence for the high reliability of the molecular weights and formulas determined by the instrument.

Figure 5d–f presents the secondary mass spectra of AFB_1_ and its two degradation products (P1 and P2), along with the corresponding structural fragment information for each substance. In the structural formulas, the red dotted line indicates the reaction cleavage site. Based on the analysis using the MassFragment tool in the MassLynx software, combined with the molecular formula information provided by the instrument, the secondary mass spectra of the three substances, and the ‘soft points’ in the AFB_1_ structure, the structures of the two degradation products were deduced.

A possible AFB_1_ degradation pathway was postulated according to the above-mentioned mass spectrometry structural analysis and identification. The results are displayed in Figure S5. AFB_1_ might first undergo a water-induced photoaddition reaction to generate photodegradation product P1, which then experienced photoreduction and photoelimination reactions, resulting in photodegradation product P2. This speculation was consistent with a photochemical reaction and the potential degradation pathway of AFB_1_ proposed in the previous paper [42]. To enhance the accuracy of our findings, future research will integrate nuclear magnetic resonance characterization to further confirm the chemical structures of P1 and P2. Additionally, an investigation into their toxicity will be conducted to provide a more comprehensive understanding of their properties and interactions.

**Figure 6:**
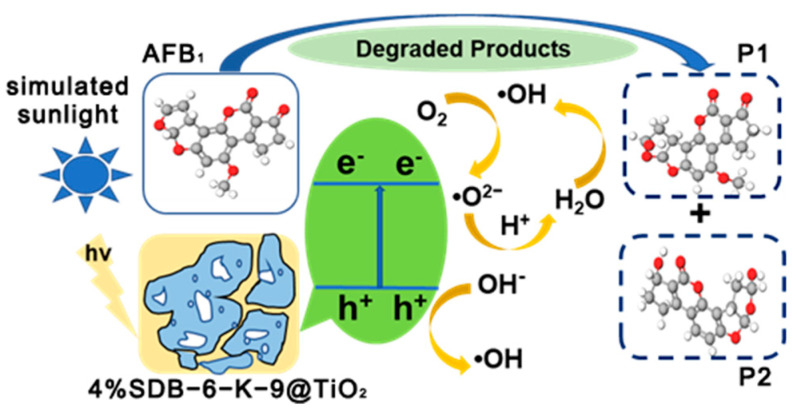
A schematic diagram illustrating the photocatalytic mechanism of AFB_1_ via the 4%SDB−6−K−9@TiO_2_ photocatalyst in simulated sunlight.

The potential photocatalytic oxidation mechanism of AFB_1_ via 4%SDB-6-K-9@TiO_2_ in simulated sunlight was postulated in Figure 6 in accordance with the results of the tests. The excited electrons produced conduction band electrons (e^−^) and valence band h^+^ when the 4%SDB-6-K-9@TiO_2_ was exposed to light. The photogenerated electrons reacted with O_2_ to generate O^2•−^ and •OH, while the h^+^ on the surface of the 4%SDB-6-K-9@TiO_2_ interacted with hydroxyl ions (OH^−^) to directly generate •OH. Some of the h^+^ and •OH oxidized the AFB_1_ adsorbed on the catalyst surface, which was consistent with previous reports [41,43,44].

## 3. Conclusions

An SDB-6-K-9@TiO_2_ composite photocatalyst is successfully prepared via hydrothermal synthesis and applied to the photocatalytic degradation of AFB_1_ in the solutions. The BET, SEM-EDS, XRD, FTIR, XPS, and UV-vis analyses show that the photocatalyst presents a high specific surface area, large pore size, and rich organic functional groups. The photocatalyst significantly improves TiO_2_ utilization for visible light. The proposed 4%SDB-6-K-9@TiO_2_ pathway and mechanism of photocatalytic AFB_1_ degradation indicate that h^+^ plays the most critical role in forming two new products after deterioration. These results show that SDB-6-K-9@TiO_2_ photocatalysis is effective and practical for degrading AFB_1_, showing potential for improving future photocatalytic treatment and promoting large-scale practical application for AFB_1_ removal.

## 4. Materials and Methods

### 4.1. Materials

The biochar SDB-6-K-9 was synthesized using soybean dreg as a carbon source. The standard AFB_1_ sample was purchased from Pribolab Biological Engineering Co., Ltd. (Qingdao, China) and stored in the dark at 4 °C. The potassium bromide, butyl titanate, hydrochloric acid, ethylene diamine tetraacetic acid, tertbutyl alcohol, p-benzoquinone, sodium hypochlorite, and chromatographic-grade barium sulfate were purchased from Sinopharm Chemical Reagent Co., Ltd. (Shanghai, China). The isopropyl alcohol was obtained from Shanghai Aladdin Biochemical Technology Co., Ltd. (Shanghai, China), while Tianjin Fuchen Chemical Reagent Co., Ltd. (Tianjin, China) provided the Tween-20 and ethanol. The chromatographic grade formic acid and isopropyl alcohol were obtained from Beijing Minrida Technology Co., Ltd. (Beijing, China), while the mass spectrometry grade acetonitrile and methanol were purchased from Fisher Co., Ltd. (Oskaloosa, IA, USA). The water used in this paper was purified using a Milli-Q system from Millipore (Billerica, MA, USA). All other chemicals and solvents were analytically pure and used as received without further purification.

### 4.2. The Preparation and Characterization of the SDB-6-K-9@TiO_2_ Composites

The simple hydrothermal synthesis method used to prepare the SDB-6-K-9@TiO_2_ composites was adapted from the available literature [21]. Here, 2 mL of a butyl titanate solution was added dropwise into 22 mL of isopropyl alcohol while magnetically stirring for 30 min. Then, 10 mL of distilled water was added to form a white suspension, which was magnetically stirred for 10 min, after which the prepared SDB-6-K-9 with different mass ratios were added under ultrasonic exposure. The homogeneous solution was transferred to a hydrothermal reactor and maintained at 180 °C for 18 h in an oven. After the reaction, the precipitated product was rinsed several times with ethanol and deionized water until the filtrate was near neutral, after which it was dried at 105 °C for 24 h and ground through a 120-mesh sieve for later use. The subsequent SDB-6-K-9@TiO_2_ composites were collected and marked as XSDB-6-K-9@TiO_2_, where X denotes the additional SDB-6-K-9 amount (1%, 2%, 3%, and 4%).

The specific surface areas and pore size distributions of the prepared SDB-6-K-9@TiO_2_ composites were obtained via the automatic specific surface of a porosity analyzer at 77 K with a relative pressure (P/P_0_) ranging from 0 to 0.99 (BET, Quantachrome, Autosorb-iQ, Boca Raton, FL, USA). The morphology and elementary composition of the samples were verified via scanning electron microscopy (SEM, ZEISS, Gemini 300, Jena, Germany) equipped with energy dispersive spectroscopy (EDS, Oxford, Xplore 30, Abingdon, UK). The X-ray diffraction (XRD) analyses were conducted via an X-ray instrument (XRD, Rigaku, Ultima IV, Tokyo, Japan) at 40 kV using Cu Kα radiation, while scanning occurred in a range of 10–80°, at a rate of 2° min^−1^. The Fourier transform infrared (FTIR) spectra of the samples were obtained using an FTIR spectrometer (FTIR, Shimadzu, Type 2000, Tokyo, Japan) in a range from 4000 cm^−1^ to 400 cm^−1^ at a resolution of 4 cm^−1^, using KBr pellets for sample preparation. The surface properties of the samples were acquired using X-ray photoelectron spectroscopy (XPS, Thermo Fisher Scientific, Escalab 250Xi, Waltham, MA, USA) with monochromatic Al Kα radiation. The UV-vis diffuse reflectance spectra (DRS) were recorded with a Model Shimadzu UV-2550 spectrometer (UV-vis, Agilent, Cary 60, Santa Clara, CA, USA).

### 4.3. The Photocatalytic Degradation Tests

The photocatalytic tests of the SDB-6-K-9@TiO_2_ composites were conducted in a photochemical reactor (Zhongjiao Keyuan, CEL-LB70, Beijing, China) [41] with a 10 cm distance between the light source and the reactor. The test procedure was as follows: 6 mg of the SDB-6-K-9@TiO_2_ composite was added to a 20 mL AFB_1_ solution at a concentration of 50 μg/mL and magnetically stirred for 120 min in a dark chamber until reaching the adsorption–analytical equilibrium. Then, the light source was turned on to initiate the photocatalytic reaction, while all of the solutions were constantly stirred during irradiation incidence. At a preset time, 1 mL of the degradation solution was centrifuged at 6000 rpm for 10 min to remove the photocatalyst and then passed through a 0.22 μm filter membrane into a brown liquid vial.

The photocatalytic tests mainly investigated the impact of different light sources (simulated daylight: 300–1100 nm and visible light: 420–800 nm), different photocatalysts (SDB-6-K-9 addition amount: 1–4%), photocatalyst dosage (3–8 mg), initial AFB_1_ concentration (50–200 μg/mL), and pH value (4–8) on the removal efficiency. All experiments were repeated three times.

The AFB_1_ reduction (Re) was calculated using the following equation:

(1) $$Re\left(\%\right)=\frac{C_{0}-C_{t}}{C_{0}}\times 100\%$$

where C_0_ (μg/mL) is the initial AFB_1_ solution concentration, and C_t_ (μg/mL) is the AFB_1_ concentration at different time points.

### 4.4. The Examination of the Photocatalytic Mechanism

Degradation experiments involving radical scavengers were conducted to examine the deterioration mechanism. Here, 1 mmol of the h^+^ (EDTA), hydroxyl radical (TBA), and superoxide radical (O_2_^•−^) scavengers (BQ) were added to the AFB_1_ solution to assess the photocatalytic degradation. All experiments were repeated three times. Furthermore, the photocatalytic reaction mechanism was explored by measuring the photocatalytic degradation products [21,41].

### 4.5. SDB-6-K-9@TiO_2_ Photocatalytic Recycling

Cyclic experiments were conducted to assess the reusability and stability of the SDB-6-K-9@TiO_2_ composite exhibiting the best degradation results. After the first photocatalytic AFB_1_ degradation process was completed in optimal photocatalytic conditions, the photocatalyst was recovered via centrifugation, washed several times with deionized water, filtered, and dried for the subsequent cycle in the same conditions. In total, five cycles were performed.

### 4.6. The Determination of the AFB_1_ and Photocatalytic Degradation Products

The AFB_1_ concentration and degradation products in the solution were determined using an Agilent-1290 UHPLC system (Agilent Technologies Inc., Santa Clara, CA, USA) with a quadrupole time-of-flight (Q-TOF) component model G6530 system (Agilent Technologies Inc., Santa Clara, CA, USA). The sample was separated using a ZORBAX Eclipse XDB C18 analytical column (50 mm × 2.1 mm, 1.8 μm) (Agilent, Santa Clara, CA, USA). The mobile phase consisted of an isometric elution of methanol/acetonitrile/0.1% formic acid in water (17.5:17.5:65, V/V/V) at a flow rate of 0.25 mL/min, a column temperature of 30 °C, and a sample injection volume of 10 μL, while the AFB_1_ concentration in the sample was quantitatively analyzed via the peak areas.

The TOF conditions consisted of an ESI positive detection mode, a capillary voltage of 3000 V, an atomizer pressure of 50 psi, a gas temperature of 300 °C, a drying gas flow rate of 10 L/min, a fragmentation voltage of 100 V, a cone h^+^ body voltage of 35 V, a scanning range of *m*/*z* 100–1000, and a scanning speed of 1 cycle/SEC. Purine (*m*/*z* 121.0508) and HP-921 (*m*/*z* 922.0097) were used as the reference solutions.

The target MS/MS mode consisted of a first-stage mass spectrometry scanning range of *m*/*z* 100–1000, with a scanning speed of 1 spectra/s, and a two-stage mass spectrometry scanning range of *m*/*z* 100–1000, with a scanning speed of 1 spectra/s. The data acquisition mode was composed of contour and bar diagrams. The degradation mechanism was inferred by comparing the obtained *m*/*z* values with the molecular weight of standard AFB_1_ and its possible conversion products.

### 4.7. Statistical Analysis

All of the measurements were conducted in triplicate, and the results are expressed as the mean *±* standard deviation. ANOVA and Duncan’s multiple comparison were performed by using the SPSS 23.0 to determine the significant differences between groups. *p* < 0.05 was considered to be significant.

## Figures and Tables

**Table 1:** The specific surface area, pore volume, and average particle size of TiO_2_ and SDB-6-K-9@TiO_2_ composites.

Sample	Specific Surface Area (m^2^/g)	Total Pore Volume (cm^3^/g)	Average Particle Size (nm)
TiO_2_	174.91 ± 6.32	0.5812 ± 0.0112	1.329 ± 0.039
1%SDB-6-K-9@TiO_2_	294.57 ± 8.61	0.5313 ± 0.0032	8.207 ± 0.075
2%SDB-6-K-9@TiO_2_	399.16 ± 4.75	0.6044 ± 0.0124	7.561 ± 0.037
3%SDB-6-K-9@TiO_2_	468.28 ± 9.46	0.6271 ± 0.0289	4.539 ± 0.029
4%SDB-6-K-9@TiO_2_	642.64 ± 10.53	0.7799 ± 0.0247	3.903 ± 0.036

## Data Availability

The data presented in this study are available on request from the corresponding author.

## Supplementary Materials

The following supporting information can be downloaded at: https://www.mdpi.com/article/10.3390/toxins16100429/s1, Figure S1: (a, e, i, m) Full spectrums; (b, f, j, n) High-resolution of C1s; (c, g, k, o) High-resolution of O1s; (d, h, l, p) High-resolution of Fe2p XPS spectras of 1%SDB-6-K-9@TiO_2_, 2%SDB-6-K-9@TiO_2_, 3%SDB-6-K-9@TiO_2_, 4%SDB-6-K-9@TiO_2_, respectively; Figure S2: The kinetic analysis for photocatalytic degradation of AFB_1_ by 4%SDB-6-K-9@TiO_2_; Figure S3: The reusability of 4%SDB-6-K-9@TiO_2_ photocatalyst for degradation of AFB1; Figure S4: The possible AFB_1_ photodegradation pathway; Figure S5: The possible AFB1 photodegradation pathway.
