# Supplementary material for: Preparation of Soybean Dreg-Based Biochar@TiO2 Composites and the Photocatalytic Degradation of Aflatoxin B1 Exposed to Simulated Sunlight Irradiation
Source: Toxins (Basel). 2024 Oct 5;16(10):429. doi: 10.3390/toxins16100429 (PMC11511473; doi:10.3390/toxins16100429)
Supplement: Supplementary file 1 [file toxins-16-00429-s001.zip › toxins-3214248-supplementary.pdf]

Supplementary Materials: Preparation of soybean dreg-based biochar@TiO_2 composites and the photocatalytic degradation of aflatoxin B_1 exposed to simulated sunlight irradiation

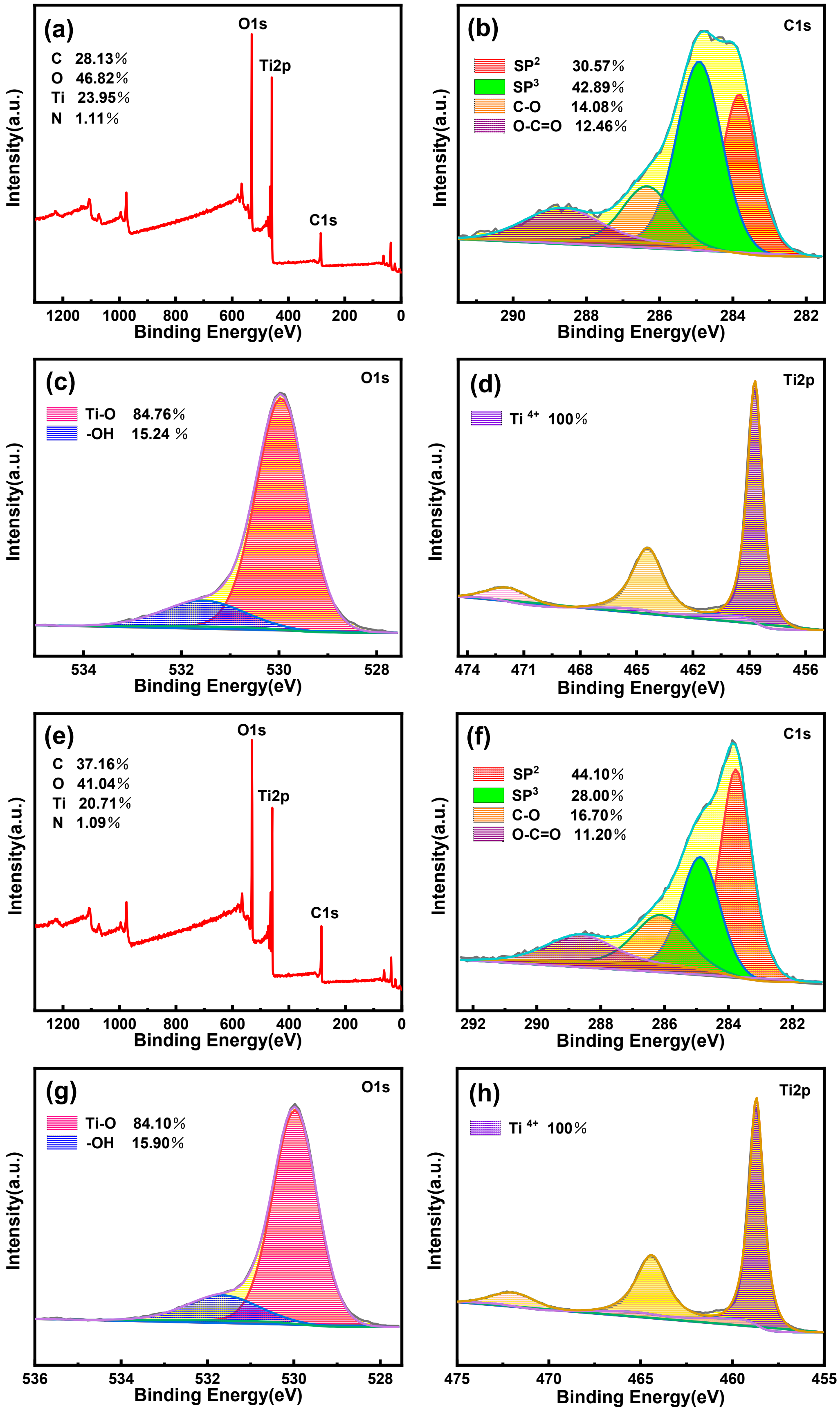
(a)
O1s
Ti2p
C1s
C 28.13%
O 46.82%
Ti 23.95%
N 1.11%
Intensity(a.u.)
1200
1000
800
600
400
200
0
Binding Energy(eV)
(b)
C1s
SP2 30.57%
SP3 42.89%
C-O 14.08%
O-C=O 12.46%
Intensity(a.u.)
290
288
286
284
282
Binding Energy(eV)
(c)
O1s
Ti-O 84.76%
-OH 15.24 %
Intensity(a.u.)
534
532
530
528
Binding Energy(eV)
(d)
Ti2p
Ti 4+ 100%
Intensity(a.u.)
474
471
468
465
462
459
456
Binding Energy(eV)
(e)
O1s
Ti2p
C1s
C 37.16%
O 41.04%
Ti 20.71%
N 1.09%
Intensity(a.u.)
1200
1000
800
600
400
200
0
Binding Energy(eV)
(f)
C1s
SP2 44.10%
SP3 28.00%
C-O 16.70%
O-C=O 11.20%
Intensity(a.u.)
292
290
288
286
284
282
Binding Energy(eV)
(g)
O1s
Ti-O 84.10%
-OH 15.90%
Intensity(a.u.)
536
534
532
530
528
Binding Energy(eV)
(h)
Ti2p
Ti 4+ 100%
Intensity(a.u.)
475
470
465
460
455
Binding Energy(eV)

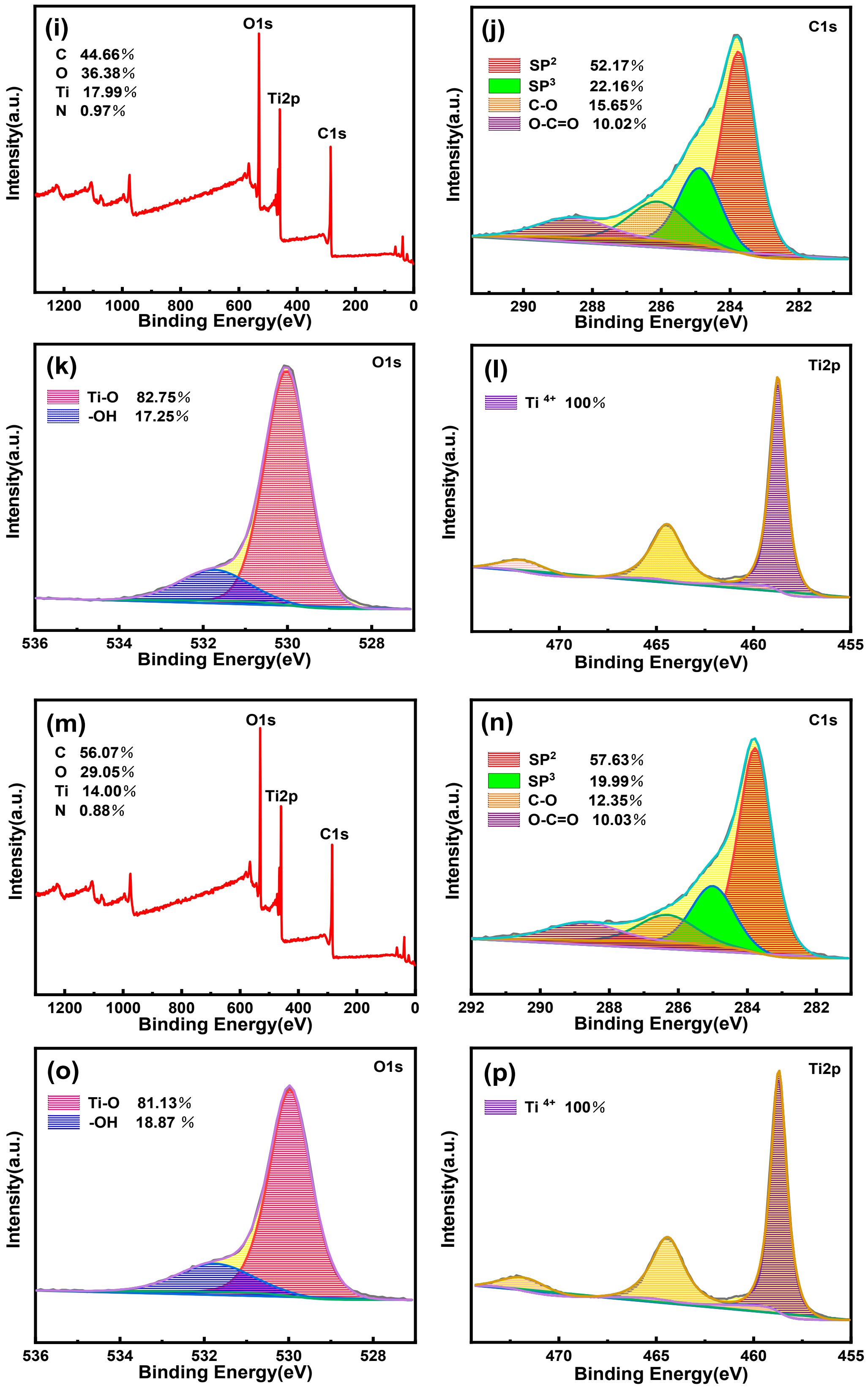

Figure S1 (a, e, i, m) Full spectrums; (b, f, j, n) High-resolution of C1s; (c, g, k, o) High-resolution of O1s; (d, h, l, p) High-resolution of Fe2p XPS spectras of 1%SDB-6-K-9@TiO_2, 2%SDB-6-K-9@TiO_2, 3%SDB-6-K-9@TiO_2, 4%SDB-6-K-9@TiO_2, respectively.

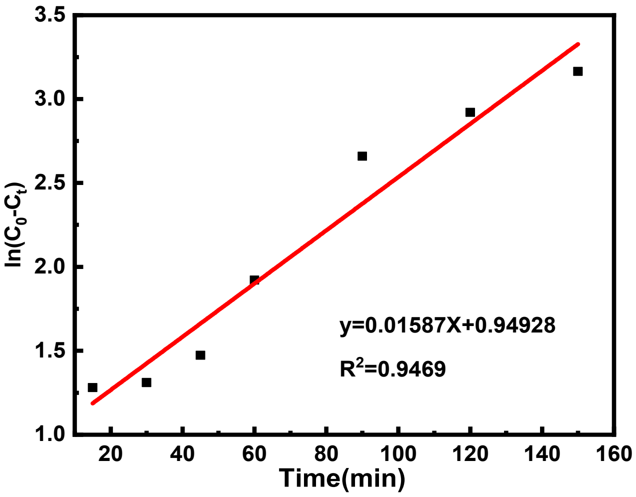

Figure S2 The kinetic analysis for photocatalytic degradation of AFB_1 by 4%SDB-6-K-9@TiO_2.

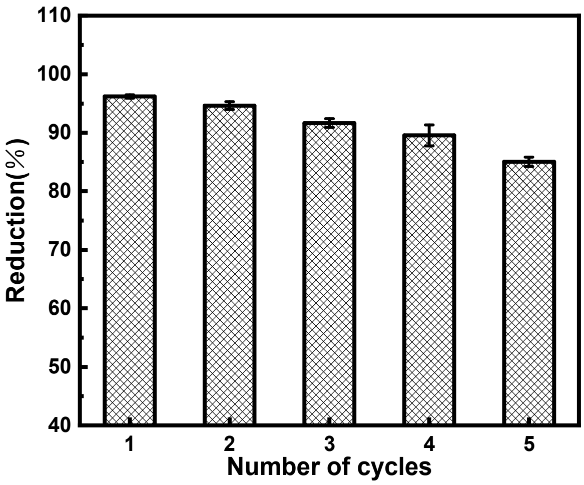

Figure S3 The reusability of 4%SDB-6-K-9@TiO_2 photocatalyst for degradation of AFB_1. The data are expressed as the mean ± standard deviation (n=3).

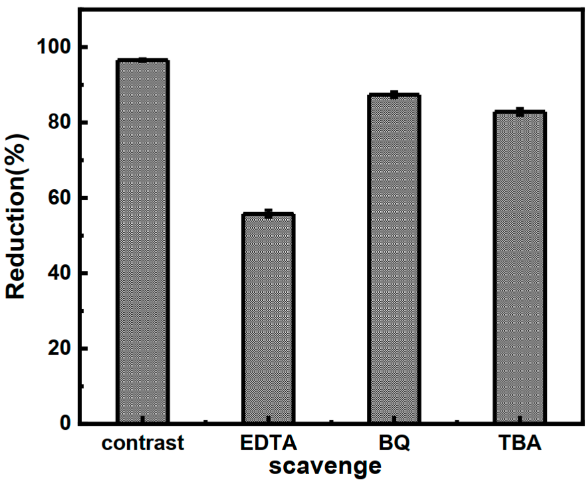

Figure S4 Effect of adding different scavengers on AFB1 removal rate. The data are expressed as the mean ± standard deviation (n=3).

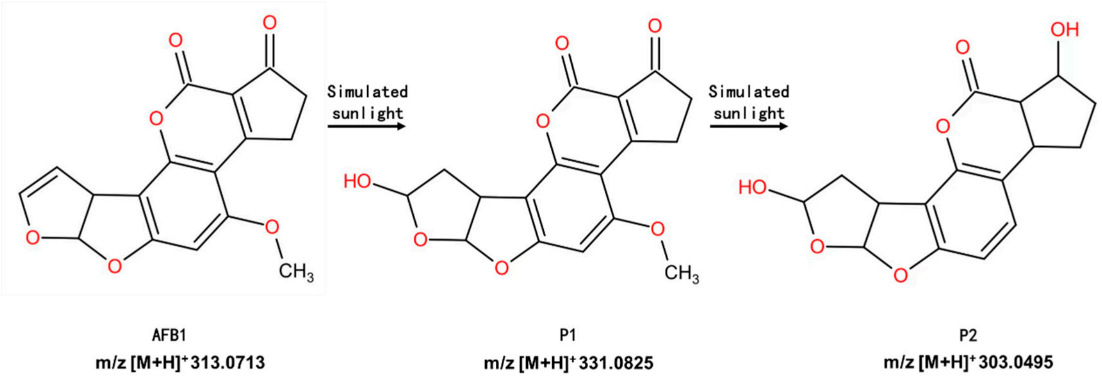

Figure S5 The possible AFB_1 photodegradation pathway.
